# Influence of baking time and formulation of part-baked wheat sourdough bread on the physical characteristics, sensory quality, glycaemic index and appetite sensations

**DOI:** 10.3389/fnut.2024.1370086

**Published:** 2024-06-03

**Authors:** Mishela Temkov, João Miguel Rocha, Cécile Rannou, Maren Ducasse, Carole Prost

**Affiliations:** ^1^Department of Food Technology and Biotechnology, Faculty of Technology and Metallurgy, Ss. Cyril and Methodius University in Skopje, Skopje, North Macedonia; ^2^CBQF—Centro de Biotecnologia e Química Fina—Laboratório Associado, Escola Superior de Biotecnologia, Universidade Católica Portuguesa, Porto, Portugal; ^3^LEPABE – Laboratory for Process Engineering, Environment, Biotechnology and Energy, Faculty of Engineering, University of Porto (FEUP), Porto, Portugal; ^4^ALiCE – Associate Laboratory in Chemical Engineering, Faculty of Engineering, University of Porto (FEUP), Porto, Portugal; ^5^ONIRIS, VETAGROBIO, UMR GEPEA CNRS 6144, Nantes, France; ^6^Biofournil, Nantes, France

**Keywords:** glycemic index, physical characteristics, satiety, sensory, sourdough bread

## Abstract

Raw materials and process parameters in bread production can modulate the glycemic index, which on itself has been linked with provision of better hunger satisfaction and maintaining better satiation. The objective of this research was to investigate if using unrefined wheat flour or the addition of intact cereals in formulation or alternating the baking time would have an effect on physical characteristics, sensory quality, glycaemic index and appetite sensations in wheat sourdough bread. In the study, three types of commercial part-baked frozen sourdough bread, baked to the final baking for two different times (long and short baking time) were used. A randomized controlled crossover trial was performed with 10 healthy adults who consumed sufficient quantity of bread to ingest 50 g available carbohydrates. Participants self-reported appetite sensations (desire to eat, hunger, fullness, satisfaction, appetite) on a 10 cm visual analog scale (VAS) scale in a course of 180 min. In addition, bread products were subjected to overall acceptability and different sensory attributes were examined on JAR “just about right” scale. Different bread formulations (refined flour, unrefined wheat flour, cereal flour or intact cereals) and different length of baking time significantly influenced (*p* < 0.005) physical, textural and sensory features of products. The alternation of aforementioned parameters decreased the glycemic index, but not significantly (*p* > 0.005). No correlation was found between lower GI, satiety and satiation. Liking score and incremental area under the curve (iAUC) of satiety and satiation were calculated as highest in sourdough bread with added cereals.

## 1 Introduction

The concept of glycemic index (GI) was developed to rank the food according to its power to raise the concentration of glucose in blood after the consumption (1). By the definition, GI corresponds to the ratio of the peak area of glucose response in blood triggered by an amount, i.e., 50 g of digestible carbohydrates present in the tested food sample and the peak area of glucose response in blood caused by the same amount of pure glucose (2). High-GI food products provoke higher insulin secretion rather than low-GI ones. In 2003, a classification system was created to effectively utilize the glycemic index (GI) values of food. According to this system, food with a GI value of 70 or higher is categorized as high-GI food. Medium-GI food falls within the range of 56 to 69, while low-GI food is classified as having a GI value of 55 or less (3). Due to evidence showing that postprandial glycemia is affected not only by the quantity but also by the quality, nature, and source of carbohydrates, the concept of glycemic load (GL) was introduced. This concept considers both the glycemic index (GI) and the carbohydrate content per serving (3–5). In this classification system, food is categorized as low (<10), medium (10–20) and high (>20) (6). Consumption of a diet high in both GI and GL on a long term is linked to the risk of developing type 2 diabetes, cardiovascular diseases or cancers (7, 8). On the other hand, low-GI diet containing dietary fibers and resistant starch might act favorably and decrease risk of obesity and some types of cancer. The supposed mechanism is through reducing the secretion of insulin, the slow release of carbohydrates in the upper gastrointestinal tract and indirectly via production of short-chain fatty acids that can regulate glucose and lipid metabolism (2, 9, 10).

White wheat bread as a source of energy is widely consumed globally on a daily basis and contributes to a large extent to the dietary high-GI. The containing starch is gelatinized during the baking process making it easily accessible to salivary and pancreatic α-amylase (3). There are multiple methods available to decrease the glycemic index (GI) of bread. These methods involve creating various barriers around the starch molecule to limit its accessibility for digestion by α-amylase, or increasing the proportion of resistant starch in the bread. To achieve low GI, wheat flour can be mixed with other cereals having higher ratio of amylose to amylopectin or incorporating in the dough network lots of intact cereal grains with insoluble fibers. Another way is to partially encapsulate starch with proteins and fibers, as it is the case with hard wheat flour for pasta. The addition of viscous soluble fibers (oat fibers, barley fibers, arabinoxylans, β-glucans) to the digestive media limit the absorption of glucose through epithelial cells, whereas the addition of organic acids (acetic, propionic and lactic acids) retard gastric emptying and straighten the linkage between starch and proteins. The fraction of resistant starch can be increased with alternating baking conditions (5, 11–13).

Sourdough bread is progressively replacing white wheat bread as it offers numerous health advantages. Sourdough fermentation generates many compounds that can improve the flavor, structure and its overall quality. Sourdough breads having lower GI reduce the demand of insulin response. There are more than a few mechanisms acting upon the change of GI. In these leavened breads, the concentration of organic acids is fairly greater due to the fermentation performed by yeasts and lactic acid bacteria (14, 15). Moreover, its density, compact crumb, less porous structure and structure cohesiveness, which are a consequence of prolonged fermentation (more than 15 h), play big role in decreasing glycemic response (11, 16). Consuming food products with a low glycemic index (GI) can have an impact on glycemia and insulinemia during the subsequent meal. These low-GI foods exhibit a prolonged absorptive phase following the initial phase, which can help regulate snacking between meals (17). Sourdough bread intake was proven to reduce some of appetite- and satiety-regulating hormones (ghrelin, GIP, and GLP-1 and produce decreased blood glucose response) (18). Moreover, sourdough fermentation plays a crucial role in reducing the formation of acrylamide in bread. Research suggest that sourdough fermentation, especially when tailored or combined with appropriate strains like *Lactobacilli*, can significantly decrease the acrylamide content in bread products. The process is acheived through gradual decrease in protein content during fermentation, which is associated with inhibiting acrylamide formation. Factors such as pH value and protein content in sourdough have been found to have a direct impact on acrylamide levels in bread, with lower pH values inhibiting the Maillard reaction pathway responsible for acrylamide formation. Additionally, the acidification rate of sourdough, influenced by strains like *Lactobacilli* that produce organic acids during fermentation, also contributes to to the reduction of acrylamide content (19–21).

Up-to-date an extensive research was done in understanding the influence of the addition of dietary fibers or intact cereals, fermentation conditions of sourdough bread or the concentration of sourdough in decreasing the GI and appetite rankings (14, 22, 23). This study seeks to explore how various potential methods for reducing the glycemic index (GI) – such as using whole meal flour with higher total dietary fiber content, incorporating intact cereals into cereal flours, or prolonging baking time – affect the overall physicochemical and sensory characteristics of three distinct commercial sourdough breads. In addition, the acceptance of different attributes in sourdough bread products was determined via hedonic and just-about-right test, which gives directional information for product reformulation. A complementary study was performed to analyze the impact of these breads on appetite sensations, such as desire to eat, hunger, fullness, satisfaction and appetite. Moreover, satiety and satiation incremental area under the curve (iAUC) was calculated to determine if any of the products would make participants feel fuller and more satisfied.

## 2 Materials and methods

### 2.1 Materials

Biofournil Factory, (France) kindly donated three types of pre-baked commercial organic sourdough breads studied in this research. The ingredient composition of different types of breads is included (Table 1).

**Table 1:** Ingredient composition of 3 types of breads.

Traditional bread (TB)	Wholemeal bread (WB)	Cereal bread (CB)
Stone ground wheat flour T65 (43%)	Stone ground wholemeal wheat flour T150 (41%)	Stone-ground cereal flours (wheat flour 34%, corn flour 1.1%, barley flour 1.1%, spelt flour 1.1%, rye flour 1.1%, buckwheat flour 1.1%, wheat gluten 0.5%) 40%
Water 32%	Water 34%	Water 32%
Old-fashioned sourdough (wheat flour T80 and water) 24%	Old-fashioned sourdough (wheat flour T80 and water) 24%	Old-fashioned sourdough (wheat flour T80 and water) 23%
Untreated sea salt 1%	Untreated sea salt 1%	Untreated sea salt 1%
		Seeds: brown flax, shelled sesame, shelled millet, shelled sunflower (3.5%), poppy seeds (0.5%)

The dough is initially kneaded in mixers with a duration of 6 min at the first speed followed by 10 min at the second speed. Following this, the dough undergoes its first fermentation stage at room temperature for about 30 min. Once fermented, the dough is portioned into individual pieces (loaves of 400 g each), based on the desired weight. After shaping, the dough undergoes its second fermentation phase lasting around 3 h and 30 min. Finally, the pre-cooking stage involves subjecting the prepared dough to a temperature of 215°C for a duration of 21 min.

The detailed nutritional and chemical composition is given in Table 2. The set of same types of three breads underwent final shorter baking (S) time of 9.5 min and longer baking time (L) of 17 min at 230°C in oven (OES 6.10 mini mobil, Convotherm).

**Table 2:** Nutritional table of sample sourdough breads.

	TB	WB	CB
Energy value (kcal/100 g) /(kJ/100 g)	248/1052	257/1085	252 /1065
Proteins (g/100 g)	7.4 ± 0.5	7.9 ± 0.5	8.2 ± 0.5
Carbohydrate (g/100 g)			
available	50.3	57.5	47.4
total	54.2	50.5	52.3
mono- and disaccharides	1.7 ± 0.7	1.5 ± 0.6	1.5 ± 0.6
total reducing	1.5 ± 0.6	1.5 ± 0.6	1.5 ± 0.6
total dietary fiber	**3.9 ± 1.4**	**7.0 ± 1.8**	**4.9 ± 1.5**
Fats (g/100 g)	1.4 ± 0.4	1.3 ± 0.4	2.5 ± 0.5
monounsaturated	0.51 ± 0.28	0.37 ± 0.24	0.83 ± 0.34
polyunsaturated	0.48 ± 0.27	0.61 ± 0.30	1.21 ± 0.40
saturated	0.35 ± 0.24	0.26 ± 0.21	0.36 ± 0.24
*trans*	<0.01	<0.01	<0.01
Ash (g/100 g)	1.64 ± 0.19	1.94 ± 0.20	1.75 ± 0.19
Salt	1.086 ± 0.109	1.215 ± 0.122	1.083 ± 0.109
Sodium (Na)	0.434 ± 0.044	0.486 ± 0.049	0.433 ± 0.044
Dry extract (g/100 g)	63.9	67.9	64.0
Mass loss on drying (g/100 g)	36.1 ± 0.6	32.1 ± 0.5	36.0 ± 0.6

Total dietary fibers are highlighted, component that is of high importance for the GI.

Pepsin from gastric mucosa (P-6887) and α-amylase from porcine origin (A-3176) were obtained from Sigma-Aldrich (Saint-Quentin Fallavier, France), whereas the amyloglucosidase from Aspergillus Niger with a specific activity of 14 U/mg protein was bought from Roche (Rotkreuz, Switzerland). D-glucose kit (ref. 103.21) was purchased from BioSenTec (Auzeville Tolosane, France). The Total Starch (AA/AMG) Assay Kit was used for the determination of total starch was bought from Megazyme.

### 2.2 Methods

#### 2.2.1 Physicochemical characterization

*Dimensions.* Bread dimensions (volume, height, width, specific volume, shape ratio, density) were measured by laser topography using a bread laser volumeter (Perten BVM-L370LC, Sweden) as AACC10-14.01 approved method (24). Specific volume and density were calculated according to Eqs. (1, 2).

(1) $$\upsilon =\frac{V}{m}$$

where ν is specific volume, V is volume and m is mass.

(2) $$\rho =\frac{m}{V}$$

where ρ is density, m is mass and V is volume.

*Color.* The color of the bread crust and crumb was evaluated with colorimeter (CR-400, Konica Minolta, Tokyo, Japan) in CIE L*a*b* system. The instrument was calibrated with white tile operating in aperture 8 mm, observer 2° and illuminant D65.

*Texture.* Texture Profile Analysis (TPA) test: three slices each of 18 mm thickness of the same bread were stacked and placed horizontally on the test table of the INSTRON 34SC-5 texturometer (Norwood, Massachusetts, United States), operated by Bluehill Universal 4.25 software. TPA test was conducted with 35 mm probe and 5 kN force load. The compression speed was at 60 mm/min, compression 40%, relaxation time 10 s, and relaxation rate at 1000 mm/min. Hardness is expressed as a force required to compress the bread between the teeth corresponding to first peak on the graph (Hardness = P_1_). Cohesiveness describes how well the bread is connected when disintegrated and it corresponds to the ratio of second and first area of peaks (Cohesiveness = $\frac{A_{2}}{A_{1}})$ . On the other hand, springiness is related to the bread freshness and tells on how much it will take the bread to return to the original form after being compressed and it relates to ratio of the distance traveled by the probe of the second bite to the distance of the first bite (Springiness = $\frac{d_{2}}{d_{1}})$ . Chewability gives information on the energy required to chew the food before it can be swallowed and it is derived as Hardness*Cohesiveness*Springiness (25).

*Moisture content.* The amount of moisture was estimated according to AACC gravimetric method 44–15.02, where roundly 2 g of product were dried at 105°C during 48 h (24).

*Water activity*. A_w_ was measured on 100 mg crumbled sample with a NOVASINA LabMaster-aw meter (Lachen, Switzerland).

*Total titratable acidity and pH.* TTA was evaluated on grounded and homogenized sourdough suspension according to AACC 02–31.01 method until pH value of 8.5 was reached while titrating it with 0.1 M NaOH (21). pH was determined on Hanna Instruments, HI 2210 pH meter (Rhode Island, US).

#### 2.2.2 Starch analysis and glycemic index

To calculate glycemic index (GI) according to the equation (3) given by Goni et al. (26) the content of total starch and the starch hydrolyzed for 90 min were determined.

(3) GI=39.21+0.803×H90

where H90 is the percentage of total starch hydrolyzed at 90 min.

To obtain total starch content in bread samples a Megazyme total starch (AA/AMG) analysis kit (Megazyme International Ireland Ltd., Wicklow, Ireland) was used.

The percentage of hydrolyzed starch at a certain time on the other hand was determined using modified procedure proposed by Iversen et al. (23). Bread-crumbled samples (50 ± 5 mg) were pre-treated with 20 mg of pepsin dissolved in HCl-KCl buffer pH 1.5 at 40°C for 60 min in magnetically stirred water bath. The solution was then adjusted to pH 7 with the addition of 30 mL 0.05 M KH2PO4, and the starch hydrolysis into maltodextrins was proceeded using α-amylase (0.1 mL, which corresponds to 2.6 U) for 90 min at 37°C. At the end, to convert maltodextrins chains into glucose, amyloglucosidase was applied (60 μL of enzyme to 1 mL of sample diluted in 3 mL of 0.4 M Na-acetate buffer pH 4.75) and the solution was incubated in magnetically stirred water bath at 60°C for 45 min. Glucose concentration was estimated using glucose enzymatic assay kit. To convert into hydrolyzed starch, glucose content was multiplied with 0.9 factor and taken as the percentage of total starch hydrolyzed for 90 min.

#### 2.2.3 Ethics statement

The sensory tests were conducted in accordance with the Declaration of Helsinki. Assessors gave written consent after reading detailed information about the study. All applicable institutional and governmental regulations concerning the ethical use of human volunteers were compiled and approved by the Faculty Committee of the Faculty of Technology and Metallurgy (Ss. Cyril and Methodius University in Skopje, Republic of North Macedonia, 09-1273/1), and the ONIRIS institution (Oniris VetAgroBio— Ecole Vétérinaire, Agroalimentaire et de l’Alimentation Nantes Atlantique—French Republic Agricultural Ministry National HighSchool, France, 23-657).

#### 2.2.4 Evaluation conditions

The study took place in the sensory analysis laboratory of Oniris (Nantes, France) which meets the requirements of the NF EN ISO 8589 (2010).

#### 2.2.5 Satiation and satiety assessment

**Figure 1:**
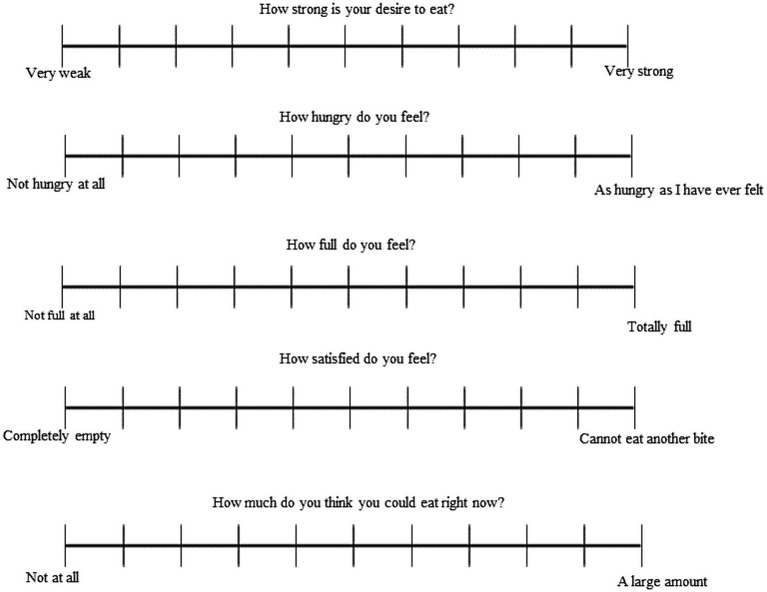
Visual analog scales for satiety assessment.

The study trials were structured for a single location and randomized through Latin Square design. Ten healthy participants with no allergies on wheat, gluten intolerance or dietary restrictions were part of the satiety assessment panel. The panel (*n* = 10) was composed of 30% males and 70% females. The participants’ ages ranged from 18 to 25 years. The Body Mass Index (BMI) distribution was of 71.9% falling within the range of 18.5 to 24.9, 10.5% within 25 to 29.9, 7% within 30 to 34.9, 1.8% below 18.5 and 8.8% who chose the “I do not want to answer” option. Regarding the panel bread consumption habits, the majority consumed bread 2 to 3 times per week (45.6%), followed by daily consumption (26.3%), once per week (19.3%), 1 to 2 times per month (7%) and less than once per month (1.8%). They were asked to follow similar food pattern the day before the visit. Participants were requested to arrive in the morning after overnight fasting for the sessions, each lasting around 3 h. Each time the participants received about 100 ± 3 grams (exact quantity calculated for each type of bread to ingest 50 grams of digestible carbohydrates) of only 1 type of test bread (TB-S, TB-L, WB-S, WB-SL, CB-S, CB-L) at a time in a random order and 300 mL of water. Participants were instructed to eat the bread within 10 min. Each participant observed his/her appetite sensations on each bread samples. During the test, participants were asked to self-report their hunger level using visual analog scales (VAS) at five different time points: before snacking, immediately after snacking, and 90, 105 and 180 min after snacking. VAS questionnaire consisted of 10 cm structured scale with ‘not hungry at all’ on the left anchor and ‘extremely hungry’ on the right anchor. The panelists were asked to assess their corresponding appetite sensations anywhere on the scale at the five designated time points using a set of five questionnaires, each comprising five scales. Then, the distance from the far – left extreme to the mark was measured and converted into numerical scores. The questionnaire is given in Figure 1.

From the data provided, satiation and satiety were calculated as integrated cumulative area under the curve over 180 min using baseline trapezoidal calculations (27).

#### 2.2.6 Sensory analysis

##### 2.2.6.1 Hedonic test

Eighty participants were asked to evaluate their liking of the 6 breads (three types of sourdough breads baked for 2 different times). The hedonic appreciation was assessed using a 9-points scale: 1 = dislike extremely; 2 = dislike very much; 3 = dislike moderately; 4 = dislike slightly; 5 = neither like nor dislike; 6 = like slightly; 7 = like moderately; 8 = like very much; 9 = like extremely. The participant demographic for this study comprised 80 individuals, with a gender distribution of 70% female and 30% male. Predominantly, their ages fell within the 18 to 25-year range (75%), 5% fell within the age range of 26 to 35 years, while 11.25% were aged between 36 and 45 years. Additionally, 7.5% were within the age range of 46 to 55 years, and 1.25% were over the age of 55.

##### 2.2.6.2 Just about right (JAR) test

Eight attributes were evaluated for each bread: toasted and cereal odor, crust color, crust crispiness, crumb softness, global aroma, acidity and humidity. Five points JAR scales were used. This scale indicates whether a particular attribute is perceived on acceptable or non-acceptable level (28, 29). The participants were instructed to determine the intensity of the attribute, whether it fell on the extreme ends (“much too low,” “not enough,” or “too high” and “too much high”) or in the middle of the scale (“just about right”).

#### 2.2.7 Statistical analysis

All results were expressed as an average ± standard deviation of different replicates at a significance level of *α* = 0.05. A two-way (formulation and baking time) analysis of variance (ANOVA) was performed for the physico-chemical measurements and a three-way ANOVA was performed for the hedonic evaluation (formulation, baking time, panel) and the satiety results (formulation, baking time, hour of intake). A post-hoc test (Least Significant Difference test) was applied when significant differences were determined. Penalty analysis was applied to the JAR results. Statgraphics Centurion XVII.I software (IBM Corp., Armonk, NY, USA) program was used for ANOVA and XLStat 2022.3.1 (Addinsoft) was used for penalty analysis and PCA.

## 3 Results and discussion

### 3.1 Physico-chemical characterization

**Figure 2:**
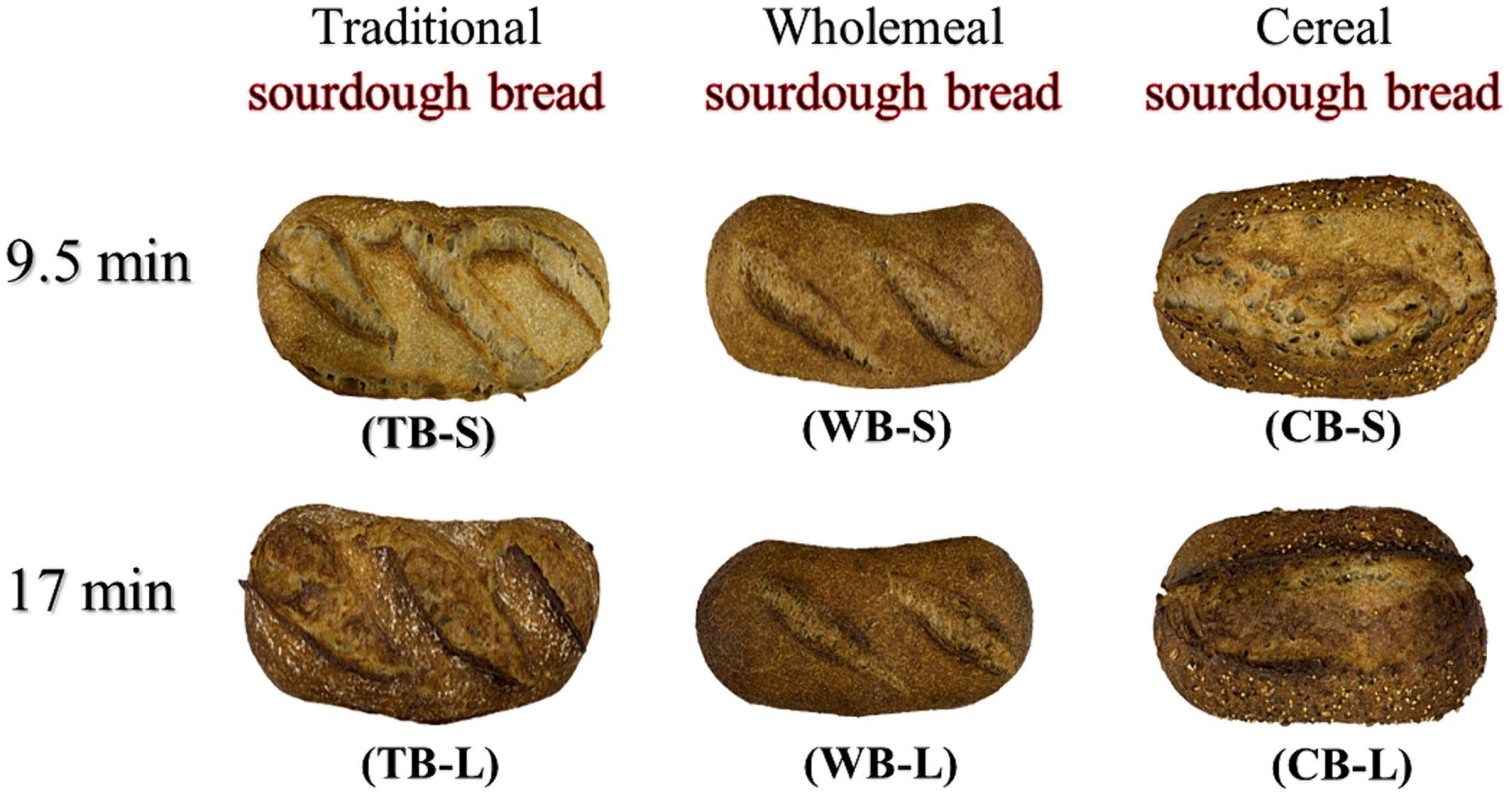
Appearance of 3 types of sourdough breads baked at 2 different times.

In the current study, three different formulations of part-baked breads (TB, WB, and CB) were baked at 230°C for 9.5 and 17 min. Their appearance is given in Figure 2. The maximum temperature reached in the center of the loaf in the first case was 79°C for the first baking time and 96°C for the second, allowing complete gelatinization of the starch. The elevated temperatures promote the breakdown of starch molecules, resulting in the transformation of starch into a form, which is more digestible and palatable. The results of the physico-chemical measurements are given in Table 3. Both formulation (_f_) and baking time (_bt_) had a significant impact (*p* < 0.05) on volume (*F*_f_ = 27.2, *p*_f_ = 0.0000; *F*_bt_ = 9.51, *p*_bt_ = 0.0081), shape ratio (*F*_f_ = 12.79, *p*_f_ = 0.0007; *F*_bt_ = 15.14, *p*_bt_ = 0.0016) and TTA (*F*_f_ = 12.77, *p*_f_ = 0.0007; *F*_bt_ = 7.86, *p*_bt_ = 0.0141). Formulation had significant influence (*p* < 0.05) on height (*F*_f_ = 3.93, *p*_f_ = 0.0443), width (*F*_f_ = 5.94, *p*_f_ = 0.0135), specific volume (*F*_f_ = 70.46, *p*_f_ = 0.0000), density (*F*_f_ = 41.69, *p*_f_ = 0.0000), pH (*F*_f_ = 252.03, *p*_f_ = 0.0000) and dry matter (*F*_f_ = 20.25, *p*_f_ = 0.0000), whereas baking time influenced significantly the mass (*F*_bt_ = 4.84, *p*_bt_ = 0.0450). The latter was primarily attributed to varying water loss throughout the distinct baking durations.

**Table 3:** Physicochemical characteristics of 3 types of bread made with sourdough, baked at two different times.

Product	V (mL)	h (mm)	w (mm)	d (mm)	SV (g/L)	ρ (g/mL)	SR	m (g)	pH	TTA (mL 0.1 M NaOH/ 10 g)	DM (%)	a_w_
TB-L	1106.0 ± 59.92 ^a/B^	240.7 ± 7.09 ^b/A^	102.0 ± 5.29 ^a/A^	89.0 ± 18.08 ^a/A^	2.44 ± 0.05 ^a/A^	0.41 ± 0.01 ^b/A^	2.75 ± 0.15 ^b/A^	453.0 ± 25.00 ^a/B^	4.313 ± 0.006 ^b/A^	6.167 ± 0.301 ^b/B^	60.03 ± 0.734 ^a/A^	0.964 ± 0.005 ^a/A^
TB-S	1155.0 ± 33.81 ^a/A^	235.0 ± 28.88 ^b/A^	100.0 ± 15.59 ^a/A^	101.3 ± 11.02 ^a/A^	2.47 ± 0.11 ^a/A^	0.41 ± 0.02 ^b/A^	2.28 ± 0.23 ^b/B^	469.3 ± 18.52 ^a/A^	4.343 ± 0.029 ^b/A^	6.933 ± 0.161 ^b/A^	59.29 ± 0.871 ^a/A^	0.963 ± 0.004 ^a/A^
WB-L	904.0 ± 73.63 ^b/B^	246.7 ± 3.06 ^a/A^	78.0 ± 12.49 ^b/A^	82.3 ± 2.08 ^b/A^	2.02 ± 0.06 ^b/A^	0.49 ± 0.02 ^a/A^	3.34 ± 0.16 ^a/A^	447.0 ± 16.64 ^a/B^	4.663 ± 0.012 ^a/A^	7.633 ± 0.355 ^a/B^	57.75 ± 0.753 ^b/B^	0.964 ± 0.002 ^a/A^
WB-S	970.0 ± 50.41 ^b/A^	266.7 ± 3.46 ^a/A^	96.0 ± 4.58 ^b/A^	98.7 ± 3.21 ^b/A^	2.03 ± 0.03 ^b/A^	0.49 ± 0.01 ^a/A^	3.01 ± 0.52 ^a/B^	477.0 ± 32.72 ^a/A^	4.653 ± 0.015 ^a/A^	7.933 ± 0.810 ^a/A^	57.98 ± 0.855 ^b/B^	0.961 ± 0.001 ^a/A^
CB-L	1071.0 ± 14.18 ^a/B^	242.7 ± 10.97 ^b/A^	104.3 ± 8.08 ^a/A^	88.3 ± 1.53 ^ab/A^	2.35 ± 0.01 ^a/A^	0.43 ± 0.01 ^b/A^	2.80 ± 0.32 ^b/A^	456.3 ± 4.93 ^a/B^	4.643 ± 0.021 ^a/A^	6.383 ± 0.275 ^b/B^	59.41 ± 0.644 ^a/A^	0.961 ± 0.005 ^a/A^
CB-S	1179.7 ± 64.05 ^a/A^	225.3 ± 1.15 ^b/A^	108.7 ± 6.66 ^a/A^	69.7 ± 10.02 ^ab/A^	2.51 ± 0.08 ^a/A^	0.42 ± 0.04 ^b/A^	2.03 ± 0.06 ^b/B^	470.0 ± 12.12 ^a/A^	4.667 ± 0.060 ^a/A^	7.117 ± 0.625 ^b/A^	59.48 ± 0.568 ^a/A^	0.963 ± 0.002 ^a/A^

V, volume; h, height; w, width; d, depth; SV, specific volume; ρ, density; SR, shape ratio; m, mass; TTA, total titratable acid; DM, dry matter. TB-L – Traditional bread, longer baking time; TB-S – Traditional bread, shorter baking time; WB-L – Wholemeal bread, longer baking time; WB-S – Wholemeal bread, shorter baking time; CB-L – Cereal bread, longer baking time; CB-S – Cereal bread, shorter baking time. Results indicate mean values ± SD. Values are the average of triplicate measurements. Data followed by different miniscule letters within a column refer to significant difference due to formulation and capital letters refer to significant difference due to baking time (ANOVA, Fisher mean square test, *p* < 0.05).

Among all the formulations, the smallest specific volume was observed for WB, which contains the highest amount of dietary fibers (Table 2). Their presence caused disruption of the gluten network with reduced gas retention capacity (13).

The specific volume declined as the baking duration increased across all formulations. This could be attributed to increased water evaporation with prolonged baking times (refer to Table 3). Since the breads were pre-baked, the initial volume and gas bubble expansion were already established. However, longer baking times are likely to result in the formation of thicker crusts, increasing internal pressure within the gas cells. This may lead to cell rupture and coalescence, resulting in reduced volume and higher density (30).

WB exhibited the highest density (0.49 g/mL) compared to TB and CB (0.41–0.43 g/mL). It is common for breads made solely from wholemeal flour to possess a dense and compact structure due to the high content of dietary fibers that are unavailable for yeast, causing fermentation and rising to cease prematurely (31).

pH and TTA are key parameters that regulate the enzymatic activity and provide insights into the ideal level of sourness in taste, respectively. During the fermentation process, the pH of sourdough bread decreases due to the predominant formation of lactic acid and acetic acid by lactic acid bacteria (LAB). It should remain within the range of 3.7–4.0, as lower values may inhibit LAB growth, while values higher than 5.8 may lead to excessive amylase activity, which could hydrolyze the starch (32). Conversely, the Total Titratable Acidity (TTA) provides information of the ratio of lactic to acetic acid, crucial for the perception of sour taste. For optimal flavor in sourdough, this ratio should ideally fall between 3:1 and 4:1. If the ratio shifts in favor of acetic acid, the taste may become overly sour (33). In the scope of the studied sourdough breads, produced organic acids led to a reduction of pH value, which varied between 4.31–4.34 for the TB and 4.64–4.67 for WB and CB. PH measures only dissociated acids, while weak acids as lactic and acetic acids do not completely dissociate. Nonetheless, lactic acid, with a pKa of 3.85, is stronger than acetic acid (pKa = 4.76), resulting in a greater dissociation of lactic acid and thus influencing the pH value more significantly. Through Total Titratable Acidity (TTA) measurements, the pH is shifted to an alkaline region, causing all acids to appear in their dissociated form, thereby reflecting their total concentration. Contrarily to pH results, WB and CB exhibited higher TTA (7.12–7.93 mL 0.1 M NaOH/10 g), as opposed to TA (6.17–6.93 mL 0.1 M NaOH/10 g) which might indicate presence of higher amount of acetic acid especially in WB. Additionally, the presence of higher amount of dietary fibers in WB create buffer, which interferes with pH. Different baking time did not affect significantly pH parameter (*F*_bt_ = 1.10, *p*_bt_ = 0.3126), but it did affect the TTA (*F*_bt_ = 7.86, *p*_bt_ = 0.0141). Breads that are baked for longer time contain lower amount of TTA. The hypothesis of this effect is associated with breakdown or degradation of some organic acids present in the dough or formation of new compounds during Maillard reactions possessing basic properties (34).

The recipe (*F*_f_ = 20.25, *p*_f_ = 0.0000) exerted significant influence on the dry matter content, while baking time did not (*F*_bt_ = 0.34, *p*_bt_ = 0.5664). Notably, WB exhibited the lowest dry matter content, measuring 57.75 ± 0.753 and 57.98 ± 0.855 for long and short baking time, respectively. This result is ascribed to the increased water adsorption capability coming from its higher dietary fiber content compared to other bread varieties, which exhibited dry matter contents ranging from 59.29 ± 0.871 to 60.03 ± 0.734.

Water activity (a_w_) ranged from 0.961 to 0.964 for all samples and was not statistically different (*p* > 0.05).

The formation of the crust color is facilitated by the moisture depletion after the crust reaches the temperature of 100°C. Below the temperature of 150°C, Maillard reactions are responsible for the color formation and above 150°C the Maillard reactions and reactions of caramelization are responsible for the browning (13, 30). The color differences might also appear from different pathways of Maillard reactions, which are pH dependent, but are also influenced by the composition of sugars and amino acids that react together (13, 35).

The color of both the crust and crumb was notably influenced by the variations in formulation and baking conditions that were examined. The most pronounced changes have happened in crust L*a*b* values as well as crumb L* values as shown in Table 4. As anticipated, breads exposed to shorter baking durations exhibited lighter shades. Prolonged baking led to increased water evaporation from the surface, accelerating the formation of melanoidins responsible for darker hues. The color variation largely depends on the specific raw materials utilized in each formulation, thus variations in color were observed among different bread formulations. With longer baking times, CB and WB displayed similar L* values on the crust (43.88 and 44.64, respectively), while TB exhibited a higher L* value (49.33). Conversely, during shorter baking, WB showed the darkest crust color (52.82), followed by CB (57.68), and TB appeared the lightest (59.70). Longer baking time increased a* value in TB formulation, but did not affect much (*p* > 0.05) WB and CB formulations. Contrarily, the baking time had a major impact on the yellowness (b* value) of the crust (*F*_bt_ = 82.61, *p*_bt_ = 0.0000). It decreased from 31.71 to 25.31, from 29.80 to 19.94 and from 30.80 to 18.92 for TB, WB and CB, respectively as the baking time extended. Among the formulations, the yellow hue was more noticeable in TB compared to the other formulations. Regarding the breadcrumb, both the lightness (L* value) and yellowness (b* value) were significantly affected by the recipe and the baking time (*F*_f_ = 50.40, *p*_f_ = 0.0000; *F*_bt_ = 31.78, *p*_bt_ = 0.0000) and (*F*_f_ = 13.27, *p*_f_ = 0.0000; *F*_bt_ = 6.49, *p*_bt_ = 0.0009), respectively. A longer baking time resulted in darker crumb coloration. Particularly, crumb color primarily depends on the raw materials used and their interactions, given that the crumb temperature remains considerably lower than that of the crust (13).

**Table 4:** Color of crust and crumb of 3 types of sourdough breads baked at two different times.

	Crumb color			Crust color		
Product	L^*^	a^*^	b^*^	L^*^	a^*^	b^*^
TB-L	64.46 ± 1.273 ^а/A^	3.31 ± 0.461 ^c/D^	16.17 ± 0.615 ^b/BC^	49.33 ± 4.354 ^a/B^	14.08 ± 1.868 ^a/A^	25.31 ± 5.844 ^a/B^
TB-S	60.28 ± 2.657 ^а/B^	3.13 ± 0.401 ^c/D^	16.06 ± 0.603 ^b/BC^	59.70 ± 3.675 ^a/A^	11.05 ± 3.388 ^a/A^	31.71 ± 1.101 ^a/A^
WB-L	57.08 ± 1.088 ^c/C^	6.57 ± 0.124 ^a/A^	17.39 ± 0.367 ^a/A^	44.64 ± 2.673 ^b/C^	14.01 ± 2.222 ^a/A^	19.94 ± 3.964 ^a/C^
WB-S	53.84 ± 2.042 ^c/D^	6.21 ± 0.401 ^a/A^	16.65 ± 0.523 ^a/AB^	52.82 ± 3.087 ^b/B^	14.02 ± 1.543 ^a/A^	29.80 ± 1.213 ^a/AB^
CB-L	60.46 ± 1.088 ^b/B^	4.57 ± 0.449 ^b/B^	16.20 ± 1.206 ^b/BC^	43.88 ± 3.395 ^ab/C^	13.78 ± 1.703 ^a/A^	18.92 ± 4.986 ^a/C^
CB-S	58.34 ± 2.087 ^b/BC^	4.04 ± 0.400 ^b/C^	15.62 ± 0.487 ^b/C^	57.68 ± 2.520 ^ab/A^	12.38 ± 1.973 ^a/A^	30.80 ± 1.950 ^a/A^

TB-L – Traditional bread, longer baking time; TB-S – Traditional bread, shorter baking time; WB-L – Wholemeal bread, longer baking time; WB-S – Wholemeal bread, shorter baking time; CB-L – Cereal bread, longer baking time; CB-S – Cereal bread, shorter baking time. Results indicate mean values ± SD. Data followed by different miniscule letters within a column refer to significant difference due to formulation and capital letters refer to significant difference due to baking time (ANOVA, Fisher mean square test, *p* < 0.05).

**Figure 3:**
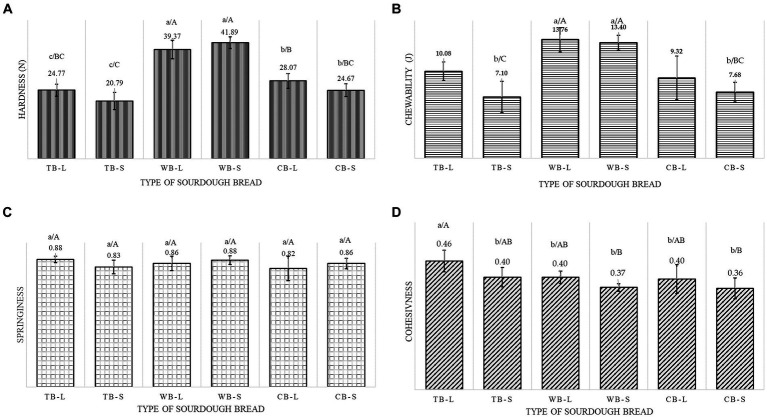
Textural properties of three types of sourdough breads baked at two different times **(A)** hardness **(B)** chewability, **(C)** springiness, **(D)** cohesiveness.

Texture in sourdough breads is highly dependent on the level of acidification. In acid conditions gluten swells, starch undergoes mild hydrolysis and in addition, short time is required for mixing the dough. Furthermore, the organic acids produced from sourdough fermentation might enhance the water uptake and enhance softness of the finished product. It might also increase the protein solubility and prevent new bond formation (36). The results of texture profile such as hardness, chewability, springiness and cohesiveness on the three examined part-baked formulations additionally baked for 9.5 and 17 min are presented at Figures 3A–D. A low value is preferred for hardness and chewability, while high values are desired for springiness and cohesiveness (37). Formulation significantly influences hardness (*F*_f_ = 118.14, *p*_f_ = 0.0000) and chewability (*F*_f_ = 38.03, *p*_f_ = 0.0000). Baking time affects hardness (*F*_bt_ = 2.61, *p*_bt_ = 0.0160) and chewability (*F*_bt_ = 9.28, *p*_bt_ = 0.0046) except for WB. This formulation required higher force (39.37 N for longer baking – 41.89 N for shorter baking) to be compressed and was the most chewable among all formulations (13.76 N for longer baking – 13.40 for shorter baking) without being affected by the baking time. This could be attributed to the increased density and less porous structure. Conversely, longer baking times for TB and CB formulations resulted in higher hardness and chewability (24.77 N and 28.07 N for TB, respectively, and 10.08 N and 9.32 N for CB, respectively) compared to shorter baking durations (20.79 N and 24.67 N for TB, respectively, and 7.10 N and 7.68 N for CB, respectively). Springiness was not affected by the formulation of the product, nor by the baking time. It was estimated in range of 0.82–0.88. Cohesiveness was found to be between 0.36 and 0.46. TB behave better when it was disintegrated due to lack of added grains and was statistically different (*F*_f_ = 8.43, *p*_f_ = 0.0011) among formulations. Baking time also affected cohesiveness statistically (*F*_bt_ = 13.78, *p*_bt_ = 0.0008), except in CB formulation. Similar results were reported by other researchers when higher quantity of dietary fibers from different sources was introduced in the sourdough bread formulation due to their interference with protein – starch network (13, 38).

### 3.2 Glycemic index, satiety and satiation

Glycemic index (GI) of the examined sourdough breads is presented in Table 5. The general assumption is that TB had higher GI than WB and CB, while longer baking time decreased the GI. However, the differences observed were not statistically significant (*F*_f_ = 2.80, *p*_f_ = 0.0946; *F*_bt_ = 0.09, *p*_bt_ = 0.7712). The highest GI of 88.09 ± 11.62 and 79.94 ± 10.40 was measured for TB-S and TB-L, respectively. When considering baking time, longer baking periods (L) led to the lowest GI values for breads enriched with added fibers (WB-L) and those made with intact cereals (CB-L), exhibiting 68.20 ± 3.13 and 68.12 ± 0.82, respectively. Conversely, shorter baking time (S) for same bread types generated higher GI values of 77.43 ± 13.97 (WB-S) and 73.56 ± 13.26 (CB-S).

**Table 5:** Satiation, satiety and glycemic index of 3 types of sourdough breads baked at two different times.

Product	Satiation (iAUC)	Satiety (iAUC)	*In vitro* GI
TB-L	761 ± 240	832 ± 192	79.94 ± 10.40^a^
TB-S	872 ± 240	897 ± 250	88.09 ± 11.62^a^
WB-L	912 ± 383	939 ± 377	68.20 ± 3.13^a^
WB-S	745 ± 357	803 ± 379	77.43 ± 13.97^a^
CB-L	863 ± 520	882 ± 545	68.12 ± 0.82^a^
CB-S	933 ± 390	978 ± 359	73.56 ± 13.26^a^

iAUC – Incremental area under the curve; GI – Glycemic index; TB-L – Traditional bread; longer baking time; TB-S – Traditional bread, shorter baking time; WB-L – Wholemeal bread, longer baking time; WB-S – Wholemeal bread, shorter baking time; CB-L – Cereal bread, longer baking time; CB-S – Cereal bread, shorter baking time.

Sourdough fermentation alone contributes to enhanced postprandial glucose and insulin responses in individuals without underlying health conditions. This effect can be attributed to the notion of an increased presence of resistant starch, which is fostered by the organic acids induced during fermentation, thereby promoting starch retrogradation (39, 40). Our results for GI for bread made with refined wheat flour and bread made with less refined flour are in line with the literature sources stating that using wholemeal flour with added bran fraction does not change the glycemic index (11). However, the presence of insoluble fibers from the bran disrupt the gluten network in bread and might facilitate its digestive process. The obtained results are in accordance with the study of Lappi et al. who confirmed that sourdough wholemeal bread induced retarded postprandial glucose and insulin responses compared with the white wheat bread, but the regular wholemeal bread had comparable postprandial response to white wheat bread (41). In addition, bread made with cereal flours incorporated with intact cereal grains also demonstrated lower GI (Table 5) which is due to the limited starch gelatinization inhibited from the fibrous network from grains, proteins, and antioxidants (11, 42, 43). The latter product in addition to reduced GI has preserved physical structure and softer texture (Figure 3A). Longer baking conditions tends to reduce the GI from 7 to 11% in the tested products. A 30% reduction of GI was reported by Stamataki et al. (39) when high amylose barley bread was baked for 20 h at 120°C instead of the conventional 45 min at 200°C owing the formation of crystalline amylose and the increase of resistant starch content. Moreover, starch gelatinization can be affected by the presence of proteins located around starch granules, which may limit granule swelling and gelatinization, contributing to reduced digestibility. In a study conducted by Reshmi et al. (42), breads incorporated with pomelo fruit (*Citrus maxima*) segments exhibited lower predicted GI and more gradual glucose release, attributed to the presence of naringin, which inhibits enzymes involved in post-prandial hyperglycemia. The effect of the presence of high content of antioxidants and flavonoids on managing blood glucose levels by decreasing hydrolysis rate of starch through inhibition of α-amylase and α-glucosidase was shown by Kareem et al. (43).

**Figure 4:**
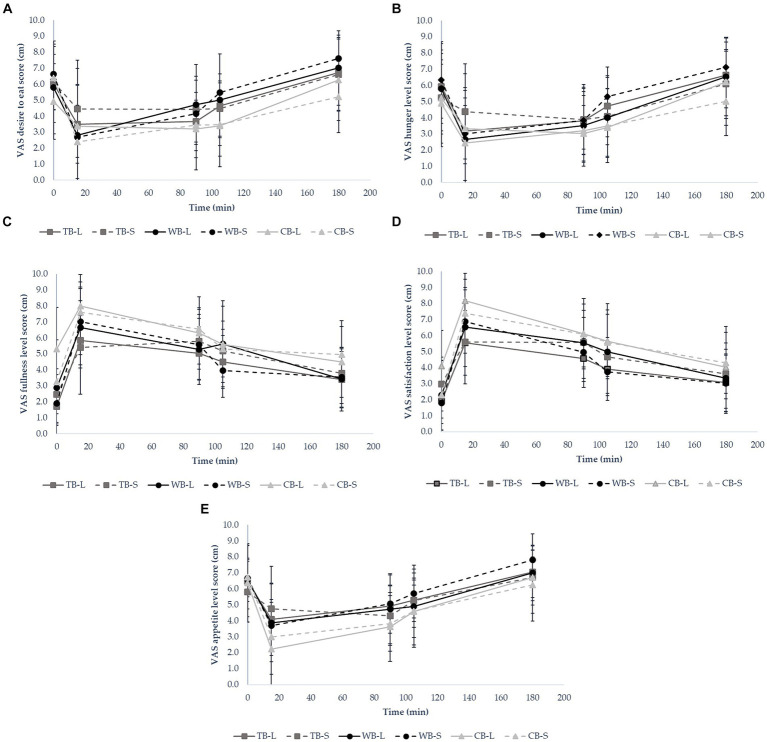
Visual analog scale (VAS) ratings of **(A)** desire to eat, **(B)** hunger, **(C)** fullness, **(D)** satisfaction **(E)** appetite over 180 min following ingestion of food (*n* = 10).

The reported visual analog scales (VAS) of postprandial satiety for tested sourdough breads (TB-L, TB-S, WB-L, WB-S, CB-L and CB-S) are plotted in Figures 4A–E. The average desire to eat, hunger and appetite scores decreased 15 min after the food ingestion, but in course of 180 min gradually increased to the initial starting level. Contrarily, the fullness and satisfaction increased immediately after eating as reported by the subjects, reaching the peak, from which point started to decline steadily until the 180 min. The desire to eat and the hunger level were the strongest after eating WB bread regardless the treatment of baking, but remained the lowest for CB-S, followed by CB-L. After food consumption, the sensation of appetite was least pronounced for CB-L, with 53% of participants reporting lower scores than TB-S and 25% than CB-S. However, at the 180-min mark post-meal, CB-S prompted a 17% reduction in average appetite compared to CB-L, and a 32% decrease compared to WB-S (Figures 4A–E). As for the desire to eat, hunger level, and appetite, TB-S initially received the highest scores, while fullness and satisfaction levels were notably lower. However, by the conclusion of the 180-min period, TB-S no longer held this position.

**Figure 5:**
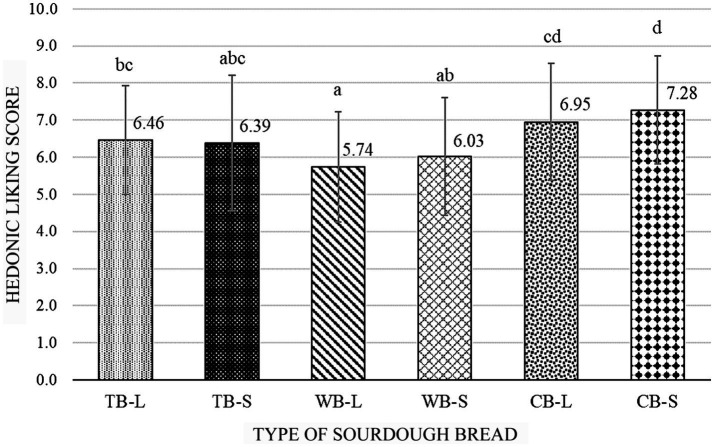
Liking score of 3 types of sourdough breads baked at two different times (*n* = 80).

The cumulative incremental area under the curve (iAUC) for satiation and satiety is presented in Table 5. Satiation represents the feeling of satisfaction, which terminates the eating, while satiety represents the feeling of fullness after food consumption both taking part in the complex system of appetite control (44). According to iAUC, satiety and satiation perceptions were the strongest for CB-S, followed by WB-L and the least pronounced for WB-S. The results show that, appetite sensations and differences in satiation and satiety perceptions have no correlation with the GI of the products, rather than the hedonic preference for it (Figure 5). However, WB-L was the least likable product, but with the lowest GI and yet demonstrated high perception of satisfaction and satiety. In literature, there are many reports that correlate low GI with better hunger satisfaction and prevent subjects from snacking in the meantime (45–47). Contrarily, there are also number of studies that do not support the above hypothesis. Andersen et al. reported no significant difference in self-reporting satiety in a study with low and high GI varieties of potatoes (48). Holt et al. found no association between GI and reported fullness of 7 types of commercial breads with equal energy portions (49). Food texture and oral processing time have a high impact on perceived satiety; harder, firmer and chewier samples being perceived as more satiating (45, 50, 51). The second highest satiety and satiation perception calculated for WB-L could be explained with the hardest and the most chewable texture besides of the lowest estimated GI.

### 3.3 Sensory analysis

#### 3.3.1 Hedonic test

Bread consumption habits of the 80 panelists included daily consumption (45%), consumption 2 to 3 times per week (45%) or once per week (10%). A significant proportion (55%) of participants showed a preference for bread that is moderately baked. Following this, a subgroup (27.5%) indicated a preference for slightly baked bread, whereas smaller segment of consumers (17.5%) expressed a liking for bread that is well-baked. Furthermore, participants exhibited preferences for various types of bread, including white bread (chosen by 51.25% of participants), baguette (preferred by 72.5%), sourdough bread (favored by 20%), wholemeal bread (preferred by 40%), cereal bread (chosen by 58.75%), gluten-free bread (selected by 1.25%), and traditional bread (preferred by 20%). Additionally, 58.75% of the participants expressed a positive preference for sourdough bread. About 33.75% showed neutrality or indifference toward this product, while a smaller subgroup of 7.5% indicated a dislike for it.

Consumer acceptability varied significantly (*F*_p_ = 10.86, *p*_p_ = 0.000) among the different types of bread evaluated individually. Additionally, the type of bread was found to have a significant influence (*F*_f_ = 25.75, *p*_f_ = 0.000) on the preferences of the panelists. However, the baking time did not have a significant effect (*F*_bt_ = 1.24, *p*_bt_ = 0.26) on consumer liking. This is evident from the comparable mean scores for TB-L and TB-S, which were 6.46 ± 1.458 and 6.39 ± 1.825, respectively (Figure 5). The highest score was recorded for the formulation CB-L (7.28 ± 1.449), followed by CB-S (6.95 ± 1.571), while WB-L and WB-S were less preferred by consumers (scoring 5.74 ± 1.490 and 6.03 ± 1.583, respectively). These results were expected considering the results obtained for hardness, chewability (Figure 3), color (Table 4) and acidity (Table 3). The less preferred product was harder to be chewed, darker in color (regardless of baking time) and considerably sourer compared to other two types of bread attributed to the higher acetic acid content. However, whole-wheat sourdough fermentation tends to improve the unfavorable sensory aspects compared to yeast fermented bakery products offering a richer flavor due to extended fermentation and intensified acidification (52–54). In a survey on a sample of 1,013 Polish consumers older than 21 years, 23.8% expressed their positive attitude toward the consumption of white bread fortified with fibers as a mean to follow healthy lifestyle, opposite of 31.1% who had neutral opinion. However, they collectively agreed that the taste of bread is more important than the health benefits to a different extent (55). Moreover, the overall acceptability of sourdough breads subjected to fermentations of 0, 3, and 6 h using different microorganisms exhibited a dependence on fermentation time, culture type, and culture dosage, both individually and in combination (56).

#### 3.3.2 “Just about right” test

In Table 6, CB-S stands out as the most favored bread, with all attributes falling within the JAR range (60–85%), except for crust crispiness (53.75%). WB-L and CB-L, which include additional cereals and fibers and have longer baking times, were perceived as too dark in color (61.25 and 67.5%, respectively). CB-L met JAR criteria for all parameters ranging from 55 to 76.25% the except the color parameter. TB-L and TB-S lacked cereal odor and global aroma for about half of the panelists, while TB-S also lacked sufficient crispy crust (55%) and toasted odor (61.25%). WB-L was deemed too dark (61.25%) and too acidic (56.25%), with low scores for crumb softness (76.25%) and crust crispiness (50%). WB-S was rated highly acidic by over half of the judges (51.9%), with only 25.32 and 35.44% finding the crumb softness and crust crispiness sufficient. Humidity met JAR standards in all products. The judges’ opinions aligned with objective results from the instrumental sensorial analysis, particularly for color and texture, which are crucial for consumer acceptance. Longer baking times darkened the surface color for all three products, but it was deemed excessive for WB and CB, whereas TB’s color was optimal for both baking times. However, short baking times did not produce enough toasted odor and global aroma (Table 6). More than 70% of the judges found the crumbs in WB-L and WB-S breads too hard which was confirmed with the texture analyzer measuring the highest values for hardness (39.37 N and 41.89 N, respectively) (Figure 3A). The acidity levels within the same product were determined to surpass the optimal range by over 50% of the evaluators. This observation could be attributed to a potentially substantial proportion of acetic acid in comparison to lactic acid, as indicated by TTA values presented in Table 3. According to the penalty analysis, a range of attributes, distinct for each product, significantly impacted consumer satisfaction by not meeting the JAR criteria. The results presented in Table 6 include only penalties from respondents whose influence exceeded the 20% threshold (57). The overall liking of TB-L product was significantly influenced (*p* = 0.029) by the absence of cereal odor, whereas for TB-S the mean drop was affected by not only cereal odor but the crumb softness as well (*p* = 0.030). The crust color of sourdough bread was detrimental parameter for CB-L, WB-S and WB-L (*p* = 0.036, *p* = 0.005, *p* = 0.037, respectively). The performance of the latter was also penalized by the intense toasted odor (*p* = 0.021). Interestingly, CB-S, which exhibited the highest overall liking score and closely aligned with the ideal product on the JAR scale, could achieve even greater appreciation if the global aroma were more robust.

**Table 6:** Penalty analysis of sourdough breads baked at two different times (*n* = 80).

(A) Comparison between TB-L and TB-S									
Attribute	Level	Consumers (%)	Overall liking	Penalty	*p*-value	Consumers (%)	Overall liking	Penalty	*p*-value
		TB-L				TB-S			
Crust color	Much too low	12.50	6.80			47.50	5.95		
	JAR	**71.25**	6.53			**52.50**	6.79	0.22	0.541
	Much too much	16.25	5.92			0.00	/		
Toasted odor	Much too low	33.75	6.15			**61.25**	6.43		
	JAR	**58.75**	6.64			38.75	6.32	0.426	0.200
	Much too much	7.50	6.50			0.00	/		
Cereal odor	Much too low	**52.50**	6.14			**47.50**	6.05		
	JAR	36.25	6.93	0.886	0.030	43.75	6.89	0.735	0.029
	Much too much	11.25	6.44			8.75	5.71		
Crumb softness	Much too low	23.75	6.84			13.75	5.82		
	JAR	**72.50**	6.43	0.970	0.028	**70.00**	6.68	−0.114	0.756
	Much too much	3.75	4.67			16.25	5.62		
Crispy crust	Much too low	42.50	6.35			**55.00**	6.48		
	JAR	**53.75**	6.46	0.022	0.957	43.75	6.40	0.006	0.986
	Much too much	3.75	7.67			1.25	2.00		
Global aroma	Much too low	**47.50**	6.26			**50.00**	5.90		
	JAR	45.00	6.78			**50.00**	6.88	0.573	0.080
	Much too much	7.50	5.83			0.00	/		
Acidity	Much too low	6.25	6.80			12.50	5.60		
	JAR	**57.50**	6.59	0.623	0.132	**57.50**	6.65	0.293	0.378
	Much too much	36.25	6.21			30.00	6.21		
Humidity	Much too low	10.00	6.62			5.00	5.75		
	JAR	**72.50**	6.41	0.622	0.159	**68.75**	6.58	−0.177	0.631
	Much too much	17.50	6.57			26.25	6.00		

(B) Comparison between WB-L and WB-S									
Attribute	Level	Consumers (%)	Overall liking	Penalty	*p*-value	Consumers (%)	Overall liking	Penalty	*p*-value
		WB-L				WB-S			
Crust color	Much too low	5.00	5.50			16.46	5.15		
	JAR	33.75	6.22	0.732	0.037	**67.09**	6.36	1.051	0.005
	Much too much	**61.25**	5.49			16.46	5.46		
Toasted odor	Much too low	22.50	5.61			46.84	6.05		
	JAR	**48.75**	6.13	0.762	0.021	**50.63**	5.93	−0.178	0.619
	Much too much	28.75	5.17			2.53	7.00		
Cereal odor	Much too low	23.75	5.95			17.72	6.29		
	JAR	**45.00**	5.97	0.427	0.205	**58.23**	5.83	−0.447	0.214
	Much too much	31.25	5.24			24.05	6.26		
Crumb softness	Much too low	**76.25**	5.66			**70.89**	5.82		
	JAR	22.50	6.00	0.339	0.399	25.32	6.45	0.586	0.154
	Much too much	1.25	6.00			3.80	6.67		
Crispy crust	Much too low	**50.00**	5.80			**64.56**	5.94		
	JAR	45.00	5.78	0.073	0.829	35.44	6.14		
	Much too much	5.00	4.75			0.00	/		
Global aroma	Much too low	37.50	5.67			40.51	6.09		
	JAR	**43.75**	5.97	0.416	0.218	**49.37**	5.95	−0.126	0.724
	Much too much	18.75	5.33			10.13	6.00		
Acidity	Much too low	11.25	5.78			6.33	5.60		
	JAR	32.50	5.77	0.044	0.896	41.77	6.12	0.186	0.607
	Much too much	**56.25**	5.71			**51.90**	5.98		
Humidity	Much too low	38.75	5.48			32.05	6.08		
	JAR	**50.00**	5.95	0.425	0.204	**50.00**	6.18	0.359	0.315
	Much too much	11.25	5.67			17.95	5.36		

(C) Comparison between CB-L and CB-S.									
Attribute	Level	Consumers (%)	Overall liking	Penalty	*p*-value	Consumers (%)	Overall liking	Penalty	*p*-value
		CB-L				CB-S			
Crust color	Much too low	1.25	8.00			10.00	7.50		
	JAR	31.25	7.52	0.775	0.036	**78.75**	7.35	0.349	0.381
	Much too much	**67.50**	6.72			11.25	6.56		
Toasted odor	Much too low	5.00	5.75			30.00	7.08		
	JAR	**55.00**	7.23	0.533	0.124	**65.00**	7.38	0.313	0.360
	Much too much	40.00	6.81			5.00	7.00		
Cereal odor	Much too low	18.75	7.20			25.00	7.25		
	JAR	**66.25**	6.98	−0.019	0.959	**61.25**	7.29	0.028	0.934
	Much too much	15.00	6.75			13.75	7.27		
Crumb softness	Much too low	37.50	6.83			21.25	7.12		
	JAR	**56.25**	7.00	0.029	0.935	**75.00**	7.30	0.100	0.791
	Much too much	6.25	7.80			3.75	7.67		
Crispy crust	Much too low	23.75	6.63			37.50	7.17		
	JAR	**58.75**	7.13	0.340	0.334	**53.75**	7.28	0.009	0.979
	Much too much	17.50	7.00			8.75	7.71		
Global aroma	Much too low	15.00	7.08			15.00	6.42		
	JAR	**61.25**	7.10	0.296	0.406	**70.00**	7.54	0.869	0.013
	Much too much	23.75	6.63			15.00	6.92		
Acidity	Much too low	11.25	7.00			8.75	6.86		
	JAR	**71.25**	7.12	0.471	0.218	**72.50**	7.38	0.442	0.225
	Much too much	17.50	6.43			18.75	7.00		
Humidity	Much too low	15.00	7.00			5.00	7.75		
	JAR	**76.25**	6.87	−0.500	0.219	**83.75**	7.18	−0.590	0.181
	Much too much	8.75	8.00			11.25	7.78		

JAR – Just about right; TB-L – Traditional bread, longer baking time; TB-S – Traditional bread, shorter baking time; WB-L – Wholemeal bread, longer baking time; WB-S – Wholemeal bread, shorter baking time; CB-L – Cereal bread, longer baking time; CB-S – Cereal bread, shorter baking time. Bold values represent the highest percentage of respondents towards respective attribute, whether is on the extreme low or high end or in the middle.

**Figure 6:**
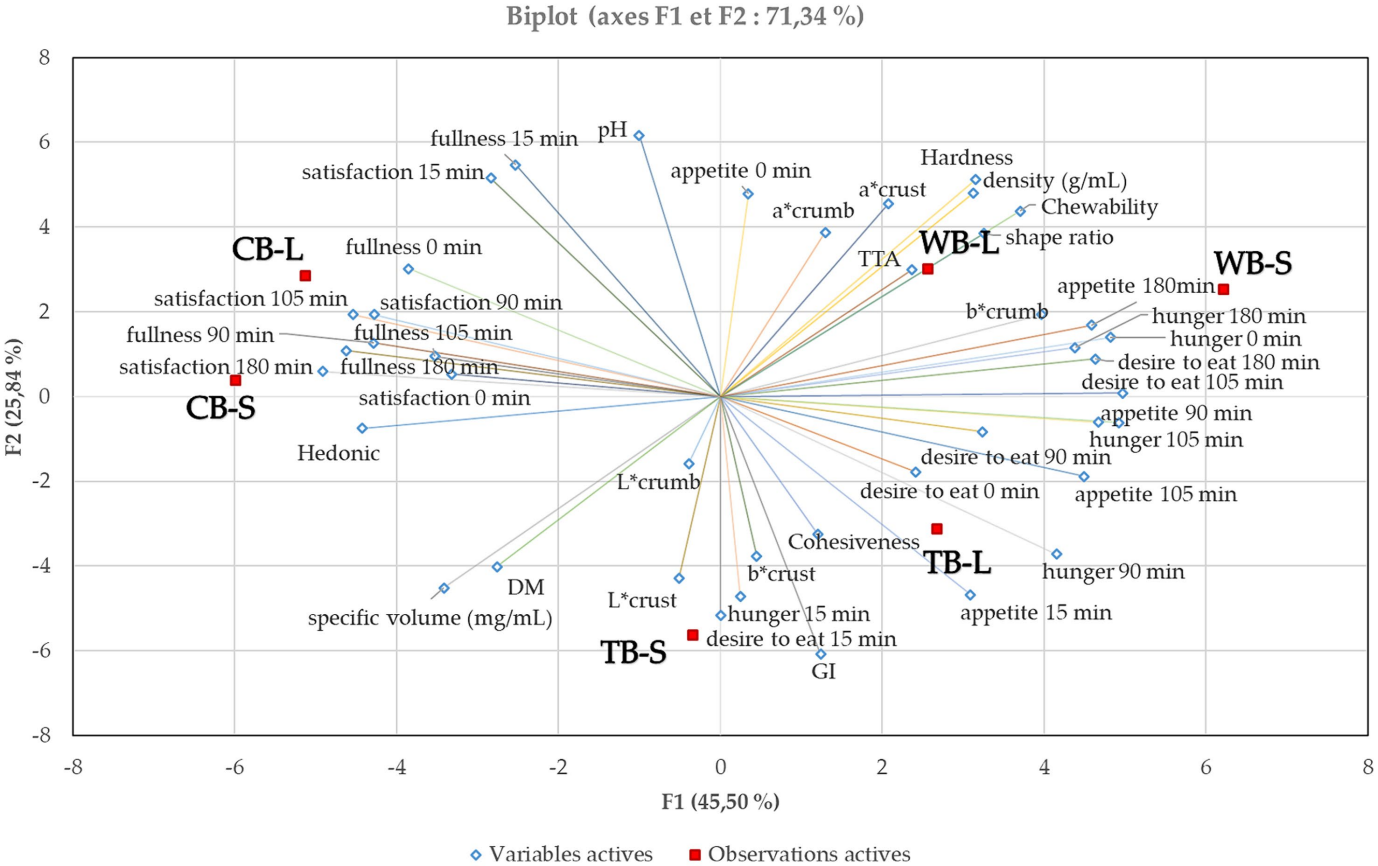
Principal Component Analysis (PCA) of the 3 different bread samples baked for two different times. The figure contains the first two principal components and their scores F1 and F2 which describe 45.5 and 25.84% of the variation within the data, respectively.

Principal component analysis was applied to investigate the combined effects of all factors employed but with reduced number of response dimensions. In this study, 42 parameters were analyzed dominated by five principal components representing 45.5, 22.8, 13.7, 7.9, and 6.9%. The loadings of the analyzed 42 responses are plotted in Figure 6.

As depicted in Figure 6, when considering not only the physical parameters but also taking into account liking scores and the induced appetite sensations over a 180-min period, the bread samples exhibit clustering by type rather than by the baking duration. Longer baking times significantly affects (*p* < 0.05) attributes such as color, mass, volume, shape ratio, and TTA, and a clear distinction among the bread types (TB, WB, and CB) is evident. Wholemeal bread is associated with the smallest shape ratio, implying greater density and consequently a more chewable and firm texture. It also correlates with higher TTA and an induction of greater appetite, desire to eat, and hunger after 180 min of consumption. These characteristics position this type of bread diagonally opposite the hedonic score, indicating a negative correlation and suggesting it was the least favored by the panelists. Conversely, CB is positively correlated with liking, satisfaction, and fullness over the 180-min period, aligning with the highest satiety and satiation scores. TB, on the other hand, is positively correlated with the lightness of both the crumb and crust, cohesiveness, and specific volume, primarily due to its absence of dietary fibers that can hinder dough proofing. Specific volume consistently shows a negative correlation with shape ratio, density, hardness, and chewability. Additionally, the sourdough bread, which lacks additional dietary fibers in the form of unrefined flour or intact cereals, exhibits the highest glycemic index (GI) but is not correlated with improved satiety or satisfaction. Moreover, the highest GI was associated with the sourdough bread that did not contained additional dietary fibers in the form of unrefined flour or intact cereals and it was not correlated with better satiety or satisfaction.

## 4 Conclusion

In the presented study, several conclusions can be drawn. To fulfill the study’s objectives, three types of part-baked commercial sourdough breads (Traditional Bread – TB, Wholemeal Bread – WB, Cereal Bread – CB) underwent baking at 230°C for either a long (L) duration (17 min) or a short (S) duration (9.5 min). Various formulations of sourdough bread, including different types of flour (wheat flour type 65, unrefined wholemeal wheat flour type 150, various cereal flours such as wheat, corn, barley, spelt, rye, and buckwheat) combined with intact whole grain cereals, along with variations in processing parameters such as baking time, have an impact on physical attributes (volume, dimensions, shape, mass, and density), chemical properties (pH, TTA, and dry matter), visual characteristics (L*a*b* values of crust and crumb), and textural qualities (hardness, elasticity, cohesion, and chewability). Different types of sourdough bread recipes, which had different quantities of total dietary fibers, and prolonged baking time did not seem to influence glycemic index significantly. No correlation was found between lower GI, satiety and satiation declarations. The self-reported appetite sensations (desire to eat, hunger, appetite) were reduced, while (fullness, satisfaction) were increased after ingestion of CB-S and CB-L immediately after eating or after 180 min. These products were also reported as the consumer favorites based on the hedonic test. Remarkably, CB-S received favorable evaluations across all attributes as “just about right,” with only the global aroma penalizing its overall liking in the JAR test. Furthermore, the incremental area under the curve (iAUC) for satiety and satiation, aside from CB-S, exhibited notable increases for WB-L, likely attributed to its harder texture and more challenging chewability during oral processing. Further research should be conducted in measuring *in vivo* GI, glucose and insulin response in blood samples in addition to VAS self-reported appetite or increase the number of panelists.

## Data availability statement

The raw data supporting the conclusions of this article will be made available by the authors, without undue reservation.

## Ethics statement

The studies involving humans were approved by Faculty Committee of the Faculty of Technology and Metallurgy (Ss. Cyril and Methodius University in Skopje, Republic of North Macedonia, 09-1273/1), and the ONIRIS institution (Oniris VetAgroBio— Ecole Vétérinaire, Agroalimentaire et de l’Alimentation Nantes Atlantique—French Republic Agricultural Ministry National HighSchool, France, 23-657). The studies were conducted in accordance with the local legislation and institutional requirements. The participants provided their written informed consent to participate in this study.

## Author contributions

MT: Writing – original draft, Visualization, Methodology, Investigation, Formal analysis, Conceptualization. JR: Writing – review & editing, Funding acquisition. CR: Writing – review & editing, Software, Methodology, Data curation. MD: Writing – review & editing, Resources. CP: Writing – review & editing, Supervision, Project administration, Methodology, Funding acquisition, Conceptualization.
